# Impact of dietary interventions in inborn errors of metabolism in paediatric dentistry: Review of the literature and case series

**DOI:** 10.1002/ccr3.3603

**Published:** 2020-12-09

**Authors:** Lorna Hirst, Suhaym Mubeen, Anupam Chakrapani

**Affiliations:** ^1^ Great Ormond Street Hospital for Children London UK

**Keywords:** growth and development, medically compromised, prevention

## Abstract

Dietary modifications in certain IEMs are highly cariogenic, subsequently posing a significant risk to dental health. Multidisciplinary input is imperative to ensure metabolic dietary needs are met, whilst complementing dental preventive regimes.

## Graphical abstract

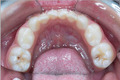

## 1 INTRODUCTION

Inborn errors of metabolism (IEM) present a heterogenous group of genetic disorders, typically characterised by a single gene defect encoding an enzyme essential for a specific metabolic pathway. Prevention of metabolic crisis often necessitates prescription of amino acid‐based formulas or other dietary modifications. Frequently, the dietary prescription is highly cariogenic and there can be a conflict of interest between maintaining dental health and supporting systemic health. This may be complicated by material risks of hypoglycaemia and the role dental infection may play in precipitating metabolic crises. The clinical cases presented in this article outline the metabolic and dietetic intervention and comprehensive dental management under general anaesthetic of patients with Medium Chain Acyl Coenzyme A Dehydrogenase Deficiency, Isovaleric Acidemia, and Maple Syrup Urine Disease, who were treated at the dental department in Great Ormond Street Hospital for Children, a tertiary referral hospital.

Inborn errors of metabolism are biochemical disorders categorized dependant on whether the metabolism of fat, protein, carbohydrates, or other complex organelles is affected.^1^, ^2^ The subsequent accumulation of toxic intermediates or sequelae of impaired synthesis of necessary compounds produces a variety of metabolic derangements (Table 1). Clinical presentation in the neonatal period is typically subtle and insidious, thus presenting diagnostic challenges even amongst astute clinicians.^3^, ^4^ Patients commonly present with nonspecific symptomatology comprising lethargy, vomiting, decreased feeding, hypoglycemia, and neurological abnormalities.^3^, ^4^ Life preserving and emergency treatment are therefore often instigated prior to formulation of a definitive diagnosis.

**TABLE 1:** Common types of IEM

Disorders relating to	Examples
Carbohydrate metabolism	Glycogen storage disorder (GSD), G6PD deficiency, hereditary fructose intolerance.
Amino acid metabolism	Maple syrup urine disease (MSUD), phenylketonuria (PKU), glutaric aciduria.
Fatty acid oxidation	Medium chain acyl coa dehydrogenase deficiency
Mitochondrial function	Kearns Sayre syndrome, Leigh syndrome
Peroxisomal function	Zellweger syndrome, Adrenoleukodystrophy
Organic acid metabolism	Isovaleric acidaemia, Propionic acidaemia, Methylmalonic acidaemia
Lysosomal storage disorders	Mucopolysaccharidosis, Gaucher disease, Niemann Pick

Delayed diagnosis and misdiagnoses in patients with IEM are frequent. This can cause anxiety and an element of uncertainty. Comparably to many childhood chronic illnesses, the multifaceted physical, psychological, and social sequelae of inborn errors of metabolism frequently result in a poorer health‐related quality of life (HRQoL).

## 2 CASE DESCRIPTIONS

This paper describes three patients aged six to eight with diagnoses of medium chain acyl coenzyme A dehydrogenase deficiency, isovaleric acidemia, and maple syrup urine disease. All patients presented with gross dental caries requiring treatment under general anesthesia. A summary of their management is presented in Table 2.

**TABLE 2:** Metabolic and dental management of the three cases

Demographics	Metabolic		Dental	Anesthetic management
	Metabolic diagnosis	Metabolic management		
Six‐year‐old female	Medium chain acyl CoA dehydrogenase deficiency (MCADD)	**Routine management** Avoidance of fasting	**Diagnoses:** Caries, Abscess	IV fluids: 10% dextrose and 0.45% NaCl until oral nutrition recommenced.
		**Emergency management** Carbohydrate polymer, for example, SOS™ ^a^ Glucogel	**Management:** Extraction of first primary molars	
			**Prevention:** Oral hygiene instruction, liaison with metabolic dietician, sealants of second primary molars, 22 600 ppm fluoride varnish	
Eight‐year‐old male	Maple syrup urine disease (MSUD)	**Routine management**	**Diagnosis:** Caries	
		Protein restriction Precursor‐free amino acid mix, for example, MSUD gel™ ^b^	**Management:** Extraction of all primary canines, primary molars and first permanent molar teeth	IV fluids: 10% dextrose, 0.45% saline, 10 mmol/KCl in 500 mL at full maintenance rates.
		**Emergency management** Stop natural protein intake Carbohydrate polymer Precursor‐free amino acid mix	**Prevention:** Oral hygiene instruction, liaison with metabolic dietician, 22 600 ppm fluoride varnish	
Six‐year‐old male	Isovaleric acidaemia	**Routine management**	**Diagnosis:** Caries	
		Protein restriction L‐Carnitine Glycine	**Management:** Extraction of 10 primary teeth (54, 51, 61, 62, 64, 65, 85, 84, 74, 75)	
		**Emergency management** Stop natural protein intake Carbohydrate polymer L‐Carnitine Glycine	**Prevention:** Oral hygiene instruction, liaison with metabolic dietician, 22 600 ppm fluoride varnish	

^a^ S‐O‐S10™ S‐O‐S15™ S‐O‐S20™ S‐O‐S25™: 10, 15, 20, and 25% carbohydrate solution (carbohydrate of which sugars at 9%).

^b^ MSUD gel™ (24 g sachets): Prescription formula of leucine, isoleucine, and valine free protein substitute (carbohydrate of which sugars at 27%).

Case 1 presents a patient with a disorder of fatty acid oxidation (medium chain acyl coenzyme A dehydrogenase deficiency [MCADD]). Individuals with MCADD exhibit an obstruction in the metabolism of fat into energy due to a deficiency in the enzyme medium chain acyl CoA dehydrogenase. Partially metabolized fatty acids accumulate in the liver, causing hepatomegaly and the brain, causing neurological symptoms.^2^ Since fats cannot be converted into an energy source, hypoglycemia materializes in the absence of adequate carbohydrate intake.^4^ Consequently, emergency metabolic regimes, such as S‐O‐S™ carbohydrate and Glucogel, are often indicated.

Medium chain acyl coenzyme A dehydrogenase deficiency represents the most common disorder of fatty acid oxidation, at a prevalence of 1 in 10 000.^2^ Presentation is often in the first or second year of life, typically during a viral illness when the metabolic demands are greater. Commonly, as in our case, patients experience multiple viral illnesses in their first few years of life, mandating an emergency regime, such as S‐O‐S™ carbohydrate and Glucogel. Empirical advice for parents emphasizes the importance of providing a glucose polymer at the first signs of being unwell, such as drowsiness or lethargy. This provides a safety barrier against subsequent hypoglycemia and deterioration of consciousness. Consequently, glucose is given at the discretion of the parent/guardian; however, it is feasible that this preventative measure could be implemented frequently in order to “err on the side of caution.”

Case 2 presents an 8‐year‐old male with maple syrup urine disease (MSUD), an autosomal recessive disorder caused by a deficiency of branched chain alpha ketoacid dehydrogenase complex.^3^ This results in accumulation of three branched chain amino acids (BCAAs): leucine, isoleucine, and valine.^1^, ^4^ MSUD gets its name from the sweet odor of the urine during metabolic crises. Elevation of leucine levels can precipitate brain injury and neurological symptoms, as shown with our patient.^4^

Maintenance of MSUD typically requires weekly blood spot branched chain amino acids (BCAA) to monitor the leucine, isoleucine, valine, and alloisoleucine. Periods of illness promote protein catabolism and subsequent increased bioavailability of branched chain amino acids. Resultant elevated leucine and branched chain keto acids levels can consequently result in rapid neurological deterioration.^5^ An emergency regime therefore requires cessation of substrate amino acids and replacement with glucose polymers.^6^ It is therefore highly plausible that in the absence of additional preventative measures, children who experience multiple metabolic relapses are at an increased risk of dental caries.

**FIGURE 1:**
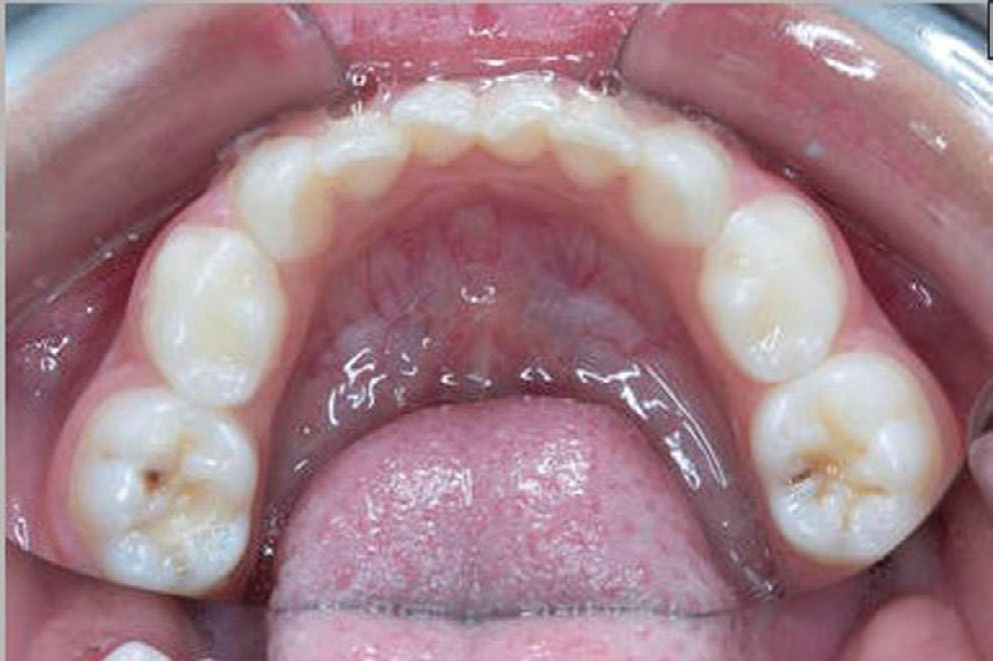
Caries experience in a patient with isovaleric acidaemia.Jpg

Case 3 (Figure 1) presents a 6‐year‐old male with isovaleric acidaemia, an autosomal recessive organic acid disorder caused by a deficiency in isovaleryl CoA which is required for leucine metabolism.^7^, ^8^ Treatment involves protein restriction and supplementation of the diet with carnitine and glycine which convert isovaleric acid into nontoxic metabolites.^7^, ^8^ During times of intercurrent illness, dietary protein restriction is required in conjunction with carbohydrate polymer, L‐Carnitine, and glycine.

## 3 DISCUSSION

Inborn errors of metabolism are uncommon, and patients are typically managed in specialist pediatric units conducive to excellent multidisciplinary care. Pediatric dentists should form an integral component of the patient's treating medical team for a multitude of reasons. Generally, their involvement provides reassurance to families that all aspects of their child's health and well‐being are being considered and safeguarded. Furthermore, prescribed dietary modifications in patients with IEM have the propensity to be highly cariogenic, thus making dental caries and infection highly probable.

Input from specialist metabolic dieticians is invaluable since protein restriction in a growing patient can be detrimental.^7^, ^9^ A conflict of interest may be present between promoting normal growth and development against safeguarding dental health. Inadequate dental preventive strategies thus increase caries risk and subsequent infection, which is a potent catabolic stressor. Consequently, a bidirectional relationship exists between dental disease and metabolic health.

Dietetically, patients with IEM present with a myriad of challenges. A restrictive lifelong dietary regime provides the mainstay in emergency management of many IEM.^10^, ^11^ To mitigate the consequential medical and developmental outcomes, strict adherence is often warranted. Demonstrably, this presents a major treatment burden, and necessitates significant modifications to both the patient's and the family's everyday life. Inborn errors of metabolism affecting protein metabolism often require protein restriction in order to control intake of the causative amino acid. Consequently, a carbohydrate and glucose rich diet may be prescribed diurnally or even nocturnally in severe cases to provide energy in growing children.^2^ Evidently, this poses a material risk to caries experience in this pediatric population.

Diagnosis of an IEM should prompt the clinician to make a timely referral to pediatric dental services for an initial assessment and implementation of a robust preventative dental regime. This should normally include liaison with metabolic dietitians, intensive oral hygiene instruction and in appropriately aged children, fluoride varnish application and fissure sealant provision. Recall intervals should reflect the potentially high caries risk and frequent dental examinations offer an opportunity for parent and patient oral health education and reinforcement of dental preventive strategies.

Where operative dental intervention is inevitable, the guiding principle in treatment planning should be to reduce the risk of future infection, a potent catabolic stressor. It may therefore be more appropriate to extract nonvital or abscessed primary molars in comparison with nonvital pulp therapy.^2^ Additionally, the clinician should carefully justify the decision to restore primary teeth with deep carious lesions and a questionable pulpal prognosis. Where restorative intervention is contemplated for early carious lesions, the durability of the restorative material should be carefully considered. Full coverage restorations may be indicated in order to reduce the risk of secondary caries and subsequent odontogenic infection.

Management of uncooperative pediatric patients with gross caries typically requires treatment under general anesthetic, which further complicates therapy. The heterogeneity of IEM combined with their low prevalence often dictates the need for specialist anesthetists.^12^ Anesthetic implications are vast and beyond the scope of this paper; however, challenges in airway management, metabolic dysregulation, seizures, and cardiac dysfunction can be observed.^12^ Perioperative management is problematic due to accelerated protein catabolism, long fasting periods, and subsequent precipitation of metabolic crisis.^7^, ^12^

The characteristically progressive and multisystemic natural history of inborn errors of metabolism undoubtedly has the predisposition to adversely impact family life. Consequently, without comprehensive and early oral health education, dental care may not be seen as a priority in families looking after a child with chronic illness. Clinically, patients may present late with unrestorable caries, odontogenic infection, and pain. Resultant treatment planning may necessitate a more aggressive approach, frequently under general anesthesia, thus posing further risk to the child's general and metabolic health.

Inborn errors of metabolism and their treatment present a multitude of physical and psychological sequelae for the patient and their families, both from lifelong dietary restrictions and from medical comorbidities such as neurocognitive disorders, hepatic, renal, and motor disorders. Collaboration across the multidisciplinary team is imperative to ensure that metabolic dietary needs are established in accordance with preventative dental advice and coordinated restorative intervention.

## CONFLICT OF INTEREST

None declared.

## AUTHOR CONTRIBUTIONS

LH: selected the cases and wrote the manuscript. SM: reviewed, edited, and provided guidance for subsequent drafts of the manuscript. AC: verified and contributed to the metabolic content of the manuscript.

## ETHICAL APPROVAL

Informed written consent was obtained from all parents in this case series prior to submission regarding the publication of images and data.

## Data Availability

Data sharing was not applicable to this article as no datasets were generated or analyzed in the production of the manuscript.

## References

[1] Marsden DL . Inborn errors of metabolism: classification and biochemical aspects In: Allen L , Prentice A , eds. Encyclopaedia of human nutrition, 3rd edn Amsterdam: Elsevier;2012;2013:1‐10.

[2] Cleary M , Francis D , Kilpatrick N . Oral health implications in children with inborn errors of intermediary metabolism: a review. Int J Pediatr Dent. 2003;7(3):133‐141. 10.1046/j.1365-263x.1997.00229.x 9482037

[3] Schillaci LP , DeBrosse SD , McCandless SE . Inborn errors of metabolism with acidosis: organic acidemias and defects of pyruvate and ketone body metabolism. Paediatr Clin North Am. 2018;65:209‐230. 10.1016/j.pcl.2017.11.003 29502910

[4] Dasgupta A , Wahed A . Clinical chemistry, immunology and laboratory quality control. Amsterdam: Elsevier; 2014.

[5] MacDonald A , Daly A , Chakrapani A . Dietary treatment of amino acids inborn errors of metabolism. Hong Kong J Paediatr. 2004;9:253‐276.

[6] Nyhan W , Rice‐Kelts M , Klein J , Barshop B . Treatment of the acute crisis in maple syrup urine disease. Arch Pediatr Adolesc Med. 1998;152(6):593‐598. 9641714 10.1001/archpedi.152.6.593

[7] Humphrey L , Kiberenge R , Nguyen T , Sobey JH , Austin T. Anaesthetic management of a patient with isovaleric acidemia. A and A Case Reports. 2015;4(3):37‐38. 25642957 10.1213/XAA.0000000000000096

[8] Chinen Y , Nakamura S , Tamashiro K , et al. Isovaleric acidaemia: therapeutic response to supplementation with glycine, L‐carnitine, or both in combination and a 10‐ year follow‐up case study. Mol Genet Metab Rep. 2017;11:2‐5. 30547004 10.1016/j.ymgmr.2017.03.002 PMC6282653

[9] Gokmen‐Ozel H , MacDonald A , Daly A , et al. Dietary practices in glutaric aciduria type 1 over 16 years. J Hum Nutr Diet. 2012;25(6):514‐519. 22845646 10.1111/j.1365-277X.2012.01269.x

[10] Camp KM . Expanding research to provide an evidence base for nutritional interventions for the management of inborn errors of metabolism. Mol Genet Metab. 2013;109:319‐328. 23806236 10.1016/j.ymgme.2013.05.008 PMC4131198

[11] Fabre A , Baumstarck K , Cano A , et al. Assessment of quality of life of the children and parents affected by inborn errors of metabolism with restricted diet: preliminary results of a cross sectional study. Health Qual Life Outcomes. 2013;11:158. 24050652 10.1186/1477-7525-11-158 PMC3848736

[12] Klosel S , Holzman RS Anaesthetic management of patients with inborn errors of metabolism. Anaesth Anal. 2017;125(3):822‐836. 10.1213/ANE.0000000000001689 27984225

